# Association between theta-band resting-state functional connectivity and declarative memory abilities in children

**DOI:** 10.1162/imag_a_00555

**Published:** 2025-05-07

**Authors:** Soléane Gander, Coralie Rouge, Anna Peiffer, Vincent Wens, Xavier De Tiège, Charline Urbain

**Affiliations:** Neuropsychology and Functional Neuroimaging Research Unit (UR2NF), Center for Research in Cognition & Neurosciences (CRCN), ULB Neurosciences Institute (UNI), Université Libre de Bruxelles (ULB), Brussels, Belgium; Laboratoire de Neuroanatomie et Neuroimagerie translationnelles (LN2T), UNI, ULB, Brussels, Belgium; Department of Translational Neuroimaging, CUB Hôpital Erasme, Hôpital Universitaire de Bruxelles (H.U.B.), ULB, Brussels, Belgium

**Keywords:** declarative memory, magnetoencephalography, resting-state functional connectivity, theta oscillations, childhood

## Abstract

Declarative memory formation critically relies on the synchronization of brain oscillations in the theta frequency band (4-8 Hz) within specific brain networks. The development of this capacity is closely linked to the functional organization of these networks already at rest. However, the relationship between theta-band resting-state functional connectivity and declarative memory abilities remains unexplored in children. Here, using magnetoencephalography, we examined the association between declarative memory performance and pre-learning resting-state functional connectivity across frequency bands in 32 school-aged children. Declarative memory was assessed as the percentage of correct retrieval of 50 new associations between non-objects and magical functions, while resting-state functional connectivity was measured through power envelope correlation of the theta, alpha, low beta, and high beta frequency bands. We found that stronger theta-band resting-state functional connectivity within occipito-temporo-frontal networks correlated with better declarative memory retrieval, while no correlation was observed in the alpha and beta frequency bands. These findings suggest that the functional brain architecture at rest, specifically involving theta-band oscillations, supports declarative memory in children. This mechanism may facilitate the subsequent rapid transformation of sensory input into visuo-semantic representations, highlighting the critical role of theta-band connectivity in early cognitive development.

## 1 Introduction

Children, particularly at school age, have to quickly learn large amounts of novel information about their environment (Hart et al., 2007). The declarative memory system is particularly at play to this aim, as it allows children to acquire and store broad conceptual representations and explicit knowledge about facts and events (Eichenbaum, 1997;Hart et al., 2007;Squire, 2004;Tulving, 2002).

Over the past decades, functional magnetic resonance imaging (fMRI) studies have characterized the brain regions underlying declarative memory processes in adults (review inKim, 2011). They demonstrated the key role of (para-)hippocampal and neocortical (e.g., prefrontal or temporo-parietal) interactions in the storage and the “binding” of declarative memory representations (Jenkins & Ranganath, 2010;Moscovitch et al., 2016;Ofen, 2012;Squire, 2004;Staresina & Davachi, 2010;Tang et al., 2018;Tulving, 2002;Vincent et al., 2006). In particular, it was suggested that hippocampo-neocortical functional connectivity (FC) processes allow the binding of*a priori*arbitrarily related elements (e.g., face- or object-name; object-function; words-meaning) into unique memory traces or*engrams*as well as their transfer into pre-existing memory systems (Cohen et al., 1997;Eichenbaum, 2001;Squire, 2004).

During childhood, these hippocampo-neocortical networks are continuously maturing (Blankenship et al., 2017;Ghetti & Bunge, 2012;Menon, 2013;Ofen, 2012;Uddin et al., 2011), in parallel with the development of complex learning and cognitive functions such as declarative memory. Studying school-aged children offers a unique opportunity to examine how functional brain architecture supports memory during a stage of development characterized by flexible and adaptable neural connections (Johnson, 2001). Unlike adults, whose memory processes depend on well-established brain networks shaped by years of experience, children rely on less specialized networks, introducing valuable variability for investigating brain–behavior relationships. Investigating these networks in typically developing children can also inform clinical research by providing critical insights into learning disabilities and neurodevelopmental disorders (Karmiloff-Smith, 1998).

Interestingly, studies have shown that, already at rest (i.e., in the absence of explicit or goal-directed task practice), the functional organization of memory-related networks could play a critical role in the development of learning abilities (Gerraty et al., 2014;Wang, LaViolette, et al., 2010). Resting-state FC (rsFC) is considered as a marker of functional brain network integrity or efficiency (Smith et al., 2009) and has been shown to be predictive of functionally related performance or abilities in adults (Schlaffke et al., 2017;Stillman et al., 2013;Wang, Negreira, et al., 2010). RsFC has been usually assessed using fMRI through the detection of interregional correlations of spontaneous blood-oxygen-level-dependent (BOLD) fluctuations, which are thought to reflect the synchronized neuronal activity and communication between distinct regions. As these networks identified at rest have been progressively shown to overlap with the patterns of activations observed in task-based (e.g., sensorimotor, language, memory) studies (Kahn et al., 2008;Vincent et al., 2006), rsFC is suggested to likely influence the extent to which brain regions can coordinate their activity during task performance (Schlaffke et al., 2017). Regarding declarative memory, few fMRI studies reported an association between interindividual variability in episodic memory performance (i.e., declarative memory for events) and rsFC across declarative memory-related regions of interest (ROI) such as the hippocampus and cortical regions (e.g., cingulate, precuneus, retrosplenial cortex, inferior parietal lobule), in adults (Persson et al., 2018;Vincent et al., 2006;Wang, LaViolette, et al., 2010;Wang, Negreira, et al., 2010). Similarly, three fMRI studies showed that hippocampal ROI-based rsFC was correlated with declarative memory abilities in pre-school-aged children (i.e., aged 4 to 6 years) and in adolescents (Geng et al., 2019;Riggins et al., 2016;Warren et al., 2021). Precisely, these studies showed that episodic memory performance is associated with hippocampal-dependent rsFC processes in networks involving the precuneus, the superior frontal gyrus (SFG), the superior temporal gyrus (STG) (Riggins et al., 2016), as well as the orbital frontal gyrus (OFG) (Geng et al., 2019) in young children and the inferior parietal lobule (IPL) in adolescents (Warren et al., 2021).

Still, none of the above-mentioned studies have investigated declarative memory-related rsFC processes using a broader brain approach, which do not constrain their purview to the study of hippocampal-dependent rsFC. In addition, due to the sluggishness of fMRI responses (Heeger & Ress, 2002;Logothetis, 2008), these past studies could not investigate theta frequency band (4–8 Hz) oscillatory activity, which has been reported as critical for learning and memory processes (Fell & Axmacher, 2011;Solomon et al., 2017;Staresina & Wimber, 2019). Accordingly, using intracranial or magnetoencephalographic (MEG) recordings, previous studies have reported the involvement of long-range theta-band coupling between medial temporal lobe (MTL) and prefrontal cortex (PFC) in successful declarative memory encoding or retrieval in adults (Anderson et al., 2010;Backus et al., 2016;Kaplan et al., 2014) and children (Johnson et al., 2022). Theta-band oscillatory coupling between fronto-temporal brain regions was thus understood as the possible electrophysiological mechanism that enables the transfer of information during memory formation.

Unlike fMRI, which provides an indirect measure of neuronal activity through BOLD activity recordings, MEG directly measures the magnetic fields generated by electric currents in the brain at the millisecond (ms) level with good spatial resolution, enabling it to analyze long-range functional brain connectivity processes in specific frequency bands (Engel et al., 2013;Hipp et al., 2012). MEG thus provides an exquisite opportunity to investigate how long-range theta-band rsFC prior to learning relates to subsequent declarative memory performance in school-aged children, as previously shown in the context of procedural learning and working memory tasks (Barnes et al., 2016;Van Dyck et al., 2021). Based on existing fMRI studies exploring the link between interindividual rsFC and declarative memory performance in young children (Geng et al., 2019;Riggins et al., 2016) and the established role of theta-band oscillations in declarative memory processes (Backus et al., 2016;Fell & Axmacher, 2011;Kaplan et al., 2014), we hereby hypothesized that stronger theta-band rsFC within temporo-frontal brain networks is associated with better declarative memory performance at school age.

## 2 Methods

### 2.1 Participants

Thirty-seven typically developing children aged from 7 to 12 years old participated in this study. All children were right handed, based on parental reports, and were native French speakers with self-reported normal or corrected vision. They had no history of neurological/psychiatric issues or learning, cognitive or language disabilities. The “Sleep Disturbances Scale for Children” (SDSC) (Bruni et al., 1996) allowed us to ensure that all children had normal sleep quality and sleep habits over the preceding months (SDSC total score < 67) and that they had maintained a regular sleep schedule for the three nights preceding the experiment (“St Mary’s Hospital Sleep Questionnaire”) (Ellis et al., 1981). Vigilance was also measured before the MEG recording sessions (see below) using the psychomotor vigilance task (PVT, 5 min-duration version) (Dinges & Powell, 1985) to control for potential variations in sustained attention abilities between participants (Chun & Turk-Browne, 2007).

Overall, five children had to be excluded from the analyses due to excessive movements during MEG recordings (n = 3), poor sleep quality (n = 1), or poor vigilance (n = 1). The final sample, therefore, included 32 school-aged children (15 females and 17 males, mean age ± SD: 10.0 ± 1.1 years) with normal sleep quality (mean ± SD: 37.7 ± 6.8) and regular sleep schedule (mean ± SD last 6 months-nights: 9.7 ± 0.6 h; night-3: 9.8 ± 0.9 h; night-2: 9.9 ± 0.9 h; night-1: 9.5 ± 0.7 h; p = 0.19).

The study was approved by the Ethics Committee of the HUB-Hôpital Erasme (Brussels, Belgium; Reference: P2018/335). Participants and their parents gave written informed consent.

### 2.2 Material and design

#### 2.2.1 Material

Complete details regarding the learning material features and task procedure can be found inUrbain et al. (2013),Urbain, De Tiège, et al. (2016), andPeiffer et al. (2020).

**Fig. 1.**
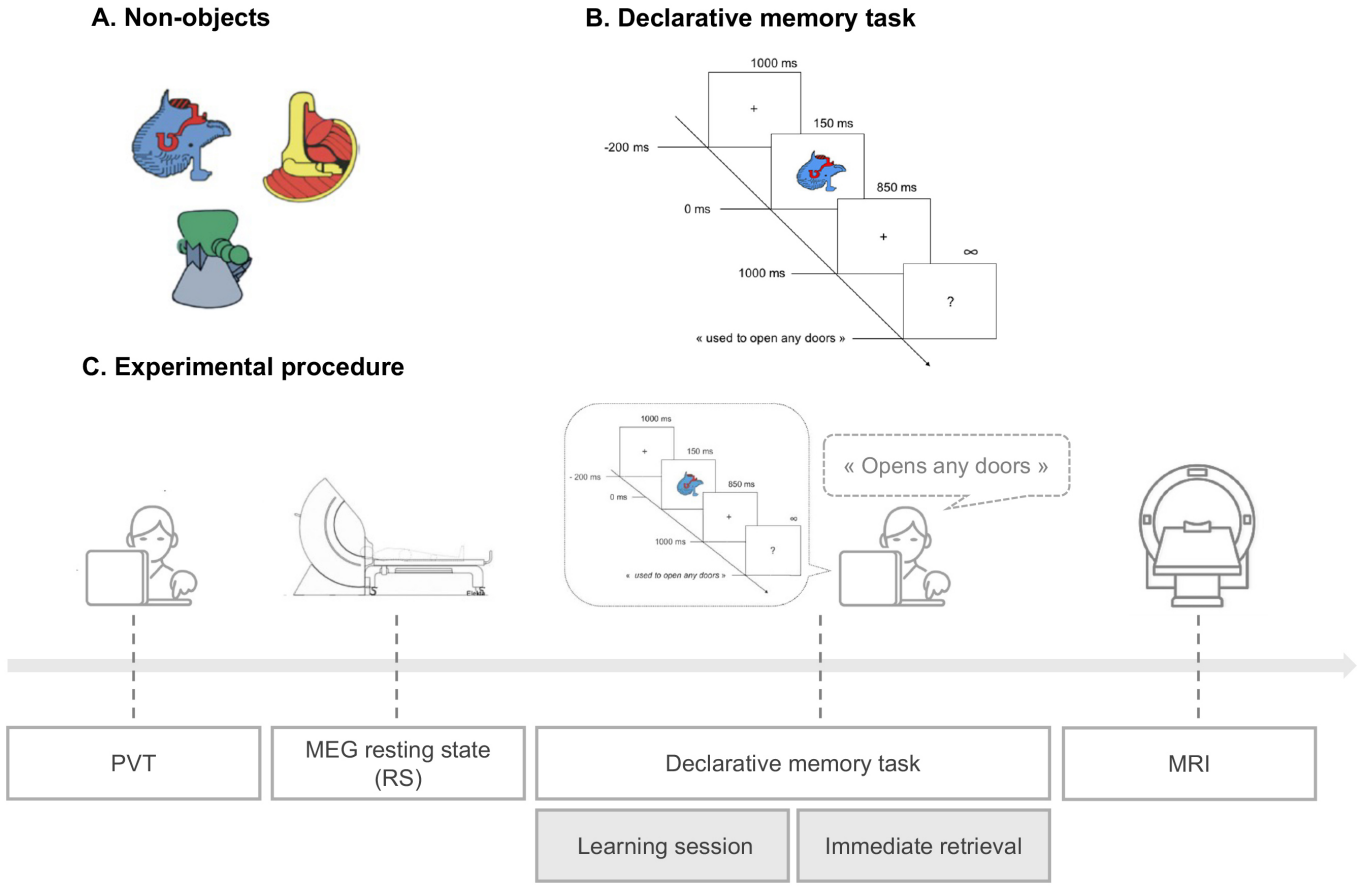
Experimental task and procedure design. (A) Sample illustrations of the 50 non-objects used. (B) Declarative memory task: At each trial, children were asked to provide the definition of the non-object presented on the screen, or skip it if the non-object was not previously defined. Responses had to be given after the appearance of a question mark (1 s after stimulus onset). (C) Experimental procedure: After a psychomotor vigilance task (PVT) and two 5-min resting-state MEG recording sessions, children underwent the declarative memory task separated into a learning session and an immediate retrieval session. A final structural high-resolution brain 3D T1-weighted magnetic resonance image (MRI) was acquired.

Briefly, 100 2D colored drawings of unknown non-objects were used in the declarative memory task (Fig. 1A). All non-objects were randomly split between 50 to-be-learned non-objects and 50 control (i.e., not learned) non-objects. Each to-be-learned non-object was randomly associated with a magical (imaginary) function that had to be learned by the participants (e.g., “paints the sky in all colors”, “opens all doors”, “stops the rain”, etc.). All definitions were in French and included three to seven words.

#### 2.2.2 Experimental design

The experimental protocol lasted half a day and occurred as follows (seeFig. 1Cfor details). Of note, this study was part of a larger research project and experimental protocol aiming at investigating various aspects of memory processes in children with typical and atypical development. Participants first performed the PVT (5 min-duration version) (Dinges & Powell, 1985), during which they had to press a response button as fast as possible each time a digital counter stimulus was presented. The mean reciprocal reaction time (RRTs = 1/Reaction Time(s)) was used as vigilance score as recommended byBasner & Dinges (2011). Then, they underwent two successive 5 min resting-state (RS) MEG recordings, which aimed at characterizing pre-learning rsFC processes in each individual. To do so, children were asked to lay down and remain as still as possible in the MEG scanner to prevent excessive motions, while focusing on a fixation cross placed on the ceiling of the magnetically shielded room. Immediately after, declarative memory performance was assessed in a behavioral session based on the declarative memory task previously developed byUrbain et al. (2013), which lasted approximately 1 h and included a 40-min learning session and a 15-min immediate retrieval session. During the learning session, children had to learn the magical functions associated with 50 non-objects (i.e., to-be-learned non-objects). The learning session included 10 learning blocks of 5 non-objects. For each learning trial, a non-object was presented on the computer screen by the experimenter who mentioned its magical function aloud to the participant. Then, each non-object was presented during 150 ms followed by 850 ms of a white screen and finally a question mark indicating to the participant to repeat the function they had just been taught. After each five non-objects, a recapitulative test (including the five non-objects) was administered. Feedback with correct responses was given to the participant during this five-by-five learning session but not during the immediate retrieval session. Participants had to reach a specific learning criterion (i.e., 60% of correctly learned associations) to ensure that all participants properly learned the material while keeping children motivated during the learning session and allowing for sufficient variability in interindividual scores. If the participant did not reach this criterion, the learning session was repeated including only the presentation of the unlearned non-objects. Once the participant reached the criterion, the immediate retrieval session started. All 50 to-be-learned non-objects and 50 control non-objects were presented twice in a random order and children had to retrieve the functions associated with each to-be-learned non-object and to correctly skip the 50 control non-objects. Each non-object was presented for 150 ms followed by an 850 ms blank screen with a fixation cross, and then a question mark prompting the child to verbally provide aloud the correct object’s function or say “skip” if unknown, with the question mark remaining on the screen until a response was given. The next trial began after a 1000-ms inter-stimulus interval corresponding to a blank screen with a fixation cross (Fig. 1B). A performance score was calculated for each participant as the percentage of to-be-learned non-objects correctly recalled during the immediate retrieval session. The protocol ended by the acquisition of a structural 3D T1-weighted brain magnetic resonance image (MRI) to allow for MEG source reconstruction.

### 2.3 Data acquisition, preprocessing, and analyses

#### 2.3.1 MEG and MRI data acquisition

MEG data (signals band-pass filtered at 0.1–330 Hz and sampled at 1 kHz) were recorded inside a magnetically shielded room (Maxshield, MEGIN, Helsinki, Finland; seeDe Tiège et al. (2008)for details) using a 306-channel whole-scalp neuromagnetometer (MEG) system (Triux, MEGIN, Helsinki, Finland) installed at the HUB–Hôpital Erasme. The head position of each participant was continuously monitored inside the MEG helmet using four head-tracking coils (Taulu et al., 2005). In addition, 3 landmark positions (left and right tragi and nasion) and at least 400 additional head-surface points (on scalp, nose, and face) were digitized using an electromagnetic tracker system (Fastrak, Polhemus, Colchester, VT, USA). MEG-compatible bipolar electrodes were used to monitor ocular, cardiac, and mouth muscle artifacts, placed vertically around the eyes (electrooculography, EOG), on the back (electrocardiography, ECG) and vertically on the chin (electromyography, EMG), respectively.

After the two 5-min resting-state MEG sessions, a structural 3D T1-weighted MRI scan was acquired in all participants (MRI, 1.5T, Intera, Philips, Best, The Netherlands) except six for whom MRI acquisition could not be performed successfully. For each of these participants, we used a linear deformation of the structural MRI of an age-matched child to best match head-surface points, using the CPD toolbox (Myronenko & Xubo Song, 2010) embedded in FieldTrip (Donders Institute for Brain Cognition and Behaviour, Nijmegen, The Netherlands, RRID:SCR_004849) (Oostenveld et al., 2011).

#### 2.3.2 MEG data preprocessing

MEG data were filtered using the temporal extension of signal space separation (tSSS; correlation coefficient, 0.98; window length, 10 s) (Taulu et al., 2005) to remove external environmental noise and correct for head movements (Maxfilter, MEGIN, Helsinki, Finland; version 2.2 with default parameters). At this stage, three subjects were excluded due to excessively noisy data. In the remaining sample, including 32 participants, no bad MEG channel was identified.

Independent component analysis (FastICA algorithm with dimension reduction to 30 and hyperbolic tangent non-linearity contrast) (Vigario et al., 2000) was applied to band-pass filtered (1–40 Hz) MEG signals to remove remaining ocular and cardiac artifacts. Components related to artifacts were visually detected and regressed out of the full rank data of each session (number of components removed: 3.5 ± 0.8, range: 2–5). The cleaned MEG data were then filtered into the theta frequency band (4–8 Hz), which was chosen for its specific role in declarative memory processes (Backus et al., 2016;Fell & Axmacher, 2011;Kaplan et al., 2014) and in the alpha (8–12 Hz), low beta (12–21 Hz), and high beta (21–30 Hz) bands to control for the specificity of the theta-band results.

#### 2.3.3 MEG source reconstruction

Participants’ structural brain MRI was preprocessed to compute the MEG forward model, which is necessary to proceed with MEG source reconstruction. The brain MRI was anatomically segmented using the FreeSurfer software (Martinos Center for Biomedical Imaging, Massachusetts, USA) (Fischl, 2012). MEG functional and brain MRI structural data were coregistered manually using the digitized fiducials and head-surface points (Mrilab, MEGIN Helsinki, Finland). A volumetric source grid (cubic with 5 mm edges) was defined in the Montreal Neurological Institute (MNI) template MRI and deformed onto each subject’s MRI using a non-linear deformation in Statistical Parametric Mapping Software (SPM12, Wellcome Trust Centre for Neuroimaging, London, UK) (Ashburner & Friston, 1999). The MEG forward model was then computed using the one-layer boundary element method implemented in the MNE-C suite (Martinos Center for Biomedical Imaging, Massachusetts, USA) (Gramfort et al., 2014).

Neuronal source activity in each frequency band was reconstructed using minimum norm estimation (MNE) (Dale & Sereno, 1993). A 10-min empty room recording preprocessed with SSS and filtered in each frequency band was used to estimate the noise covariance matrix. The MNE regularization parameter was determined using the consistency condition described in a previous study (Wens et al., 2015). The resulting three-dimensional dipole time series were further processed by projecting them onto their direction of maximum variance and obtaining their analytic signal via Hilbert transformation, as previously described bySjøgård et al. (2019)andWens et al. (2014).

#### 2.3.4 Functional connectivity analyses

**Fig. 2.**
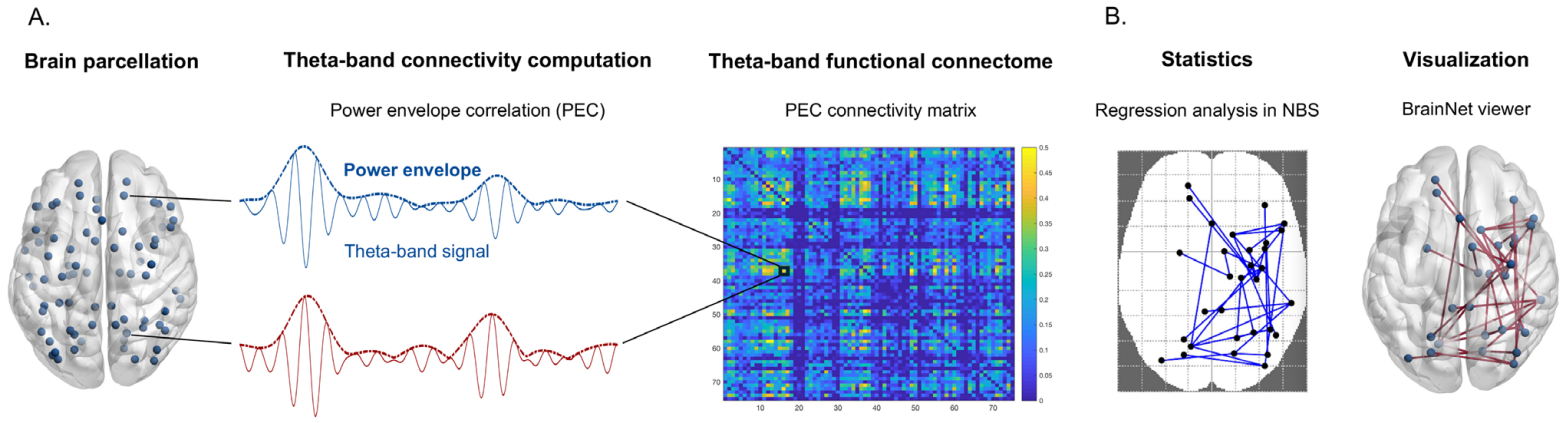
Functional connectivity and statistical analyses. (A) Neuronal theta-band source signal was reconstructed for 75 nodes from a customized parcellation of the human brain. Theta-band rsFC between each pair of nodes was calculated through power envelope correlation (PEC) of the theta-band source signals. The resulting 75-by-75 connectivity matrices were calculated for each subject. (B) The link between interindividual rsFC and behavioral retrieval performance was computed for each connection in the functional brain connectome, using a regression analysis implemented in Network-Based Statistic (NBS). Significant brain networks were then visualized using the BrainNet Viewer Connectivity Toolbox.

Theta-band rsFC between each pair of sources was calculated through power envelope correlation (Brookes et al., 2011;Hipp et al., 2012;Wens et al., 2014) after orthogonalization in order to correct for spatial leakage (Brookes et al., 2012). The power envelope correlation technique involves correlating the power envelopes of neural oscillatory time courses in the frequency band of interest (i.e., theta frequency band) of two spatially separate brain sources. This connectivity measure has previously been recognized for its ability to identify functional resting-state networks as it reliably detects the same networks widely observed with fMRI (Brookes et al., 2011;de Pasquale et al., 2010;Liu et al., 2010), while also providing frequency specificity. In fact, power envelope correlation reflects the temporal coordination in the spontaneous bursting of transient neural oscillations (Cordier et al., 2024;Seedat et al., 2020). This makes power envelope correlation particularly suited to the investigation of brain networks associated with neural oscillations such as theta oscillations. To control for the specificity of the theta frequency band connectivity in declarative memory processes, this procedure was repeated for other frequency bands showing classical brain functional networks (i.e., alpha, low beta, and high beta bands;Hipp et al., 2012;Wens et al., 2014). Power envelopes were low-pass filtered at 1 Hz before being correlated (Hipp et al., 2012). The temporal correlation between each pair of the envelope signals was calculated separately for each resting-state MEG recording (5 min) and then averaged over the two sessions to improve the stability of the power envelope correlation estimation, as recommended in previous studies (e.g., seeLiuzzi et al., 2017). This measure of connectivity was estimated at the connectome level (i.e., for each pair of nodes) using a customized parcellation of the human brain, including 75 nodes (MNI coordinates of all nodes can be found in memory atlas developed byPeiffer et al. (2021)). This parcellation involves 47 nodes from the Automated Anatomical Labeling (AAL) atlas (Tzourio-Mazoyer et al., 2002) and 28 additional nodes distributed over the whole brain that were selected from the literature for their partial or specific role in declarative memory processes (Bastin et al., 2019;Ranganath & Ritchey, 2012;Takashima et al., 2009;Urbain, De Tiège, et al., 2016). The resulting 75-by-75 connectivity matrices were calculated for each subject and for each frequency band yielding an image of their functional connectome in the frequency bands of interest (Fig. 2A). Of note, no node averaging procedure was applied nor needed given the intrinsic smoothness of MEG source reconstruction (i.e., spatial leakage; seeWens, 2015;Wens et al., 2015). To prevent potential asymmetries that could arise after pairwise orthogonalization, we followed the approach described inHipp et al. (2012)and symmetrized the matrices by taking their average with their own transpose. To exclude a potential impact of power on functional connectivity estimates (Muthukumaraswamy & Singh, 2011), we calculated the variance of the source signal at each node with depth bias correction by noise standardization (Pascual-Marqui, 2002). The influence of signal power from each node pair on the power envelope correlation of the correction connection was suppressed alongside other covariates of no interest (see below) using multivariate regression modeling.

#### 2.3.5 Statistics and reproducibility

Behavioral declarative memory performance scores were calculated for each participant as the percentage of learned non-objects correctly recalled during the immediate retrieval session. We checked for outliers considered here as exceeding three median absolute deviations from the median declarative memory performance score (Leys et al., 2013), removing them from the analyses where appropriate. We also examined whether there were correlations between declarative memory performance, age, and the children’s vigilance (RRTs) using Pearson correlations run inJamovi 2022(Version 2.3).

We used a regression analysis to infer statistical relationships between behavioral declarative memory retrieval performance and MEG pre-learning theta-band rsFC for each connection across the connectome. To test the specificity of the theta frequency band, we also replicated these regression analyses with alpha-, low beta- and high beta-bands rsFC. Performance score was inserted as covariate of interest. Age, sex, and number of trials needed for each participant to reach the 60% learning criterion were used as covariates of no interest and were regressed out of the analysis alongside node signal power (see above). The vigilance score (i.e., RRTs) was further added post hoc as covariate of no interest in order to check for potential confounding contribution of vigilance performance to actual declarative memory retrieval performance. A regression analysis with vigilance as the covariate of interest was also performed to confirm the findings of this post hoc analysis. Positive regression coefficients indicated that higher rsFC was associated with better behavioral performance, while a negative regression coefficient indicated that higher rsFC was associated with poorer behavioral performance. The statistical significance of the regression coefficients was assessed using the non-parametric Network-Based Statistic (NBS) toolbox (Zalesky et al., 2010). NBS allowed us to identify network components, that is, contiguous sets of brain connections with significant correlation between rsFC and behavioral performance, while keeping control of the Family-Wise Error Rate (FWER). An FWER-corrected*p*-value was assigned to each network component using permutation testing (n = 10000). A conservative univariate threshold of t ≥ 4.5 was applied on the rsFC analyses, ensuring that only the most robust and statistically significant connections were considered, thereby minimizing the risk of false positives and increasing the reliability of the results. Significant brain networks (i.e., associated with*p*_corr_< 0.05) were then visualized using the BrainNet Viewer Connectivity Toolbox (Xia et al., 2013) (Fig. 2B).

## 3 Results

### 3.1 Behavioral performance related to declarative memory and vigilance

No participant was identified as a behavioral outlier based on declarative memory performance score (i.e., percentage of correct responses for the learned non-objects). During the immediate retrieval session, children successfully recalled 64 ± 13% (mean ± SD percentage of correct responses) of the functions associated with the 50 to-be-learned non-objects. The number of trials needed to reach the criterion (60%) in the learning session was on average two and did not exceed three.

One child was excluded from the analyses due to excessively low vigilance (score exceeding three median absolute deviations from the median RRT score). The mean RRT over all children was 2.70/s (SD = ± 0.32/s).

No significant correlation was found between vigilance (RRTs) and declarative memory performance (Pearson correlation test,*p*> 0.3), nor between age and declarative memory performance or vigilance (Pearson correlation test,*p*> 0.3).

### 3.2 Link between theta-band resting-state functional brain connectivity and declarative memory performance

Regression analyses revealed significant positive correlations between pre-learning theta-band rsFC and subsequent declarative memory performance that emerged within two, mainly right-lateralized, neural networks (NBS,*p*_corr_*s*< 0.02). The first network included five connections among six nodes, namely connections between the right superior occipital gyrus and the medial superior frontal gyrus, the right medial prefrontal cortex, the left orbital frontal gyrus and the right amygdala, and a connection between the medial superior frontal gyrus and the right inferior temporal gyrus (*p_corr_*= 0.001). The second network included a connection between the anterior part of the right inferior temporal gyrus and the right fusiform gyrus (*p_corr_*= 0.019; see all components regrouped inFig. 3A, upper panel for the axial view and lower panel for the sagittal view, and the scatter plots for each significant connection inFig. 3B). No negative correlation was found.

**Fig. 3.**
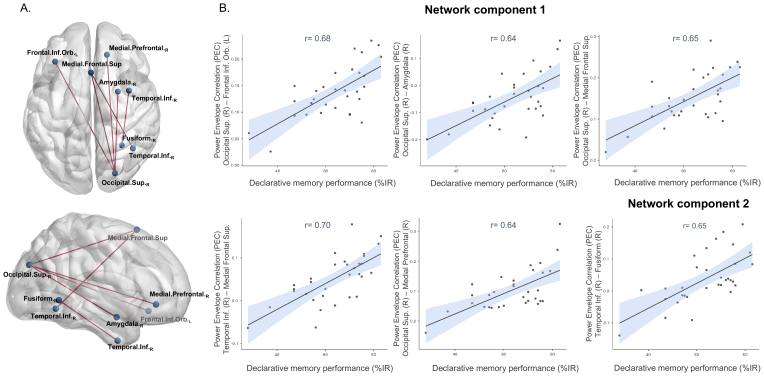
Significant correlations between pre-learning theta-band resting-state functional connectivity and subsequent declarative memory performance. (A) Connections positively correlating with declarative memory performance, represented on the MNI brain (viewed from the top (up) or the right (down)) with nodes obtained from the customized parcellation. These plots were realized using the BrainNet Viewer Connectivity Toolbox (Xia et al., 2013). (B) Scatter plots for each significant connection from the two network components. L/R = left/right hemisphere, %IR = percentage of correctly recalled objects during immediate retrieval.

Repeating these analyses (post hoc) with vigilance score as additional covariate of non-interest, we observed that the significant positive correlations between pre-learning theta-band rsFC and subsequent declarative memory performance were reduced to two significant connections (*p*_corr_*s*= 0.021) including the right superior occipital gyrus connected to the left orbital inferior frontal gyrus, and the right inferior temporal gyrus connected to the medial superior frontal gyrus. Worth noticing, this latter result was similar to the result obtained with our initial analysis when the NBS threshold was reduced (t < 4.5), suggesting that vigilance is not a primary contributor to the declarative memory-related networks. This was further confirmed by the absence of significant correlation between vigilance score and theta-band rsFC (NBS,*p*_corr_> 0.05).

Complementary regression analyses in the alpha (8–12 Hz), low-beta (12–21 Hz), and high-beta (21–30 Hz) frequency bands revealed no significant correlation between rsFC and declarative memory performance (NBS,*p*_corr_*s*> 0.05), suggesting that declarative memory performance is specifically related to theta-band rsFC.

## 4 Discussion

This study aimed at better understanding how the functional architecture of the developing brain, sustained by theta-band cortical oscillations at rest, is associated with subsequent declarative memory abilities at school age.

Results showed that 7- to 12-year-old children with stronger theta-band rsFC within two mainly right-lateralized occipito-temporo-frontal networks were also better at retrieving newly learned declarative memory information. More precisely, better declarative memory performance was associated with stronger theta-band rsFC between (i) the right superior occipital gyrus, the medial and the left orbito-frontal cortices, the right amygdala and the right inferior temporal gyrus, but also between (ii) the right inferior temporal gyrus and the right fusiform gyrus. Altogether, these findings show that the strength of offline theta-band connections between specific brain network nodes influences subsequent declarative memory performance in children.

While this study establishes a link between the functional organization of the resting brain of school-aged children and declarative memory performance, similar associations have been observed with procedural memory at school age (Van Dyck et al., 2021). Still, this past study revealed different underlying networks sustained by neural oscillations in other frequency bands. It was shown, using MEG, that procedural learning performance correlated with stronger pre-learning alpha-band rsFC within an interhemispheric sensorimotor network, encompassing the bilateral inferior parietal cortices and the primary somatosensory and motor cortices. In contrast, our study showed that declarative memory performance was associated with stronger theta-band rsFC in two mainly right-lateralized occipito-temporo-frontal networks, suggesting a dissociation of underlying neural correlates between memory domains in the resting brain in school-aged children. Supporting the hypothesis of a specific resting brain network for declarative memory abilities, our results also indicate that the theta-band rsFC processes were not directly related to attentional abilities, as no significant correlation was observed between theta-band rsFC and the vigilance score.

Among other connections, better declarative memory performance was associated with stronger rsFC between the right fusiform gyrus and the anterior part of the right inferior temporal gyrus in our study. As temporal areas are known to contribute to high-level processing and to the recognition of visual information (Geng et al., 2019), these results suggest that stronger functional connections between these regions at rest may have supported a better recognition of the learned non-objects during subsequent declarative memory retrieval in our participants. This is in line with the known functional role of the right inferior temporal gyrus in the short-term storage and recognition processes of visual inputs, which allows comparing the incoming visual information with stored memory representations (Foxe et al., 2020;Ranganath et al., 2004) as well as of the right fusiform gyrus, which facilitates the transformation of the processed sensory input (e.g., visual input) into internal to-be-stored representations through the ventral stream of extra-striate visual systems (Kim, 2011). Moreover, increases in bilateral fusiform gyri activity have been frequently reported in the context of declarative memory tasks in adults, particularly for successful encoding that helps later memory recognition processes (Garoff et al., 2005;Ofen, 2012). Hence, our results suggest that stronger rsFC within the right postero-anterior temporal areas may have improved the processing, as well as the encoding and/or the recognition of visual declarative memory information in children.

This study also revealed that better declarative memory performance was associated with stronger theta-band rsFC between bottom-up striate visual processing and top-down (pre)frontal brain areas. Among these top-down (pre)frontal regions, whose recruitment is known to depend on the content of the memory task (Wagner et al., 2001), rsFC involved the left orbito-frontal cortex (OFC) connected to the right superior occipital gyrus, the latter being mostly involved in the early stage of visual processing (Foxe et al., 2020;Geng et al., 2019). This finding aligns with previous studies reporting a role of the orbito-frontal regions in providing a rapid global estimate of the stimulus and its top-down control processing within relevant visual occipital regions (Bar, 2003;Bar et al., 2006;Engel et al., 2001;O’Shea & Walsh, 2006). Interestingly, this process may have been strengthened by the parallel functional connections of the right amygdala with the superior occipital gyrus observed in our results. Being particularly sensitive to rewarding or emotionally salient stimuli (Bar, 2003), we suggest that occipito-dependent brain processes related to the OFC and the amygdala support more efficient visual processing of learned information and, consequently, better interindividual declarative memory performance. One could thus interpret that, from a phylogenetic perspective, these top-down mechanisms may have been progressively integrated into rsFC processes to enable the subsequent rapid transformation of salient sensory inputs into internal representations and their comparison with pre-existing memory traces.

The declarative memory task used in our study not only required the recognition of visual information (i.e., non-object) but also triggered the retrieval of associated episodic and semantic information (i.e., the non-object’s magical function). This dual requirement is consistent with our results showing that a better declarative memory performance was associated with stronger rsFC between occipital-temporal regions and medial (pre)frontal regions (i.e., the right mPFC and the superior medial frontal gyrus), which play a key role in the top-down access of semantic representations (Bokde et al., 2001;Jackson, 2021;Whatmough et al., 2002). Accordingly, our results suggest that stronger theta-band mPFC-dependent rsFC may have helped children in successfully retrieving the semantic (i.e., magical functions) information associated with the visual stimulus (i.e., non-object). This interpretation is also supported by previous fMRI and MEG studies that have linked increased activity in mPFC and occipital-temporal regions to the optimal retrieval of semantic information, including information about an object’s function in children and adults (Urbain et al., 2013;Yee et al., 2010). However, it cannot be claimed that these connectivity patterns exclusively support the retrieval of associated semantic functional representations, as other episodic elements may have been learned at the same time (e.g., the context of the personal experience that accompanied the learning), possibly reflecting an interplay between episodic and semantic memory retrieval processes (De Brigard et al., 2022).

Worth noticing, despite the verbal semantic contribution of our declarative memory task, which has often been described as mainly relying on the left hemisphere (Binder et al., 2009), the resting-state brain networks associated with declarative memory performance in our study were predominantly right-lateralized. This lateralization is most likely due to the nature of our learning task requiring to bind idiosyncratic associations between novel visual, complex non-objects and their imaginary function and aligns with previous studies reporting rather right- than left-hemispheric processing in the context of complex picture learning tasks (Golby et al., 2001;Wagner et al., 1998), and especially in new situations for which no previous representation is available in long-term memory (Goldberg & Podell, 1994,^1995^;Urbain et al., 2013). Indeed, in this study, non-objects were used to reduce potential inter-individual differences in prior semantic knowledge, allowing us to investigate the link between rsFC processes and newly learned (i.e., idiosyncratic) associations. However, it should be noted that this type of learning may not fully reflect typical daily learning experiences of school-aged children. Nonetheless, acquiring idiosyncratic associations between novel objects and their functions plays a crucial role in the development of semantic representations, onto which children can later map lexical words (Clark, 2004;Jaswal, 2006), an essential process in children’s language development.

Altogether, our results suggest that declarative memory performance in children is supported by specific offline occipito-temporo-frontal theta-band functional brain connectivity processes, which may facilitate the encoding and the retrieval of new complex visuo-semantic representations (Staresina & Wimber, 2019). However, it is important to acknowledge certain limitations in this study. First, due to the correlational nature of our research design, we cannot establish causal relationships between rsFC and declarative memory performance. Secondly, while the implementation of a learning criterion (i.e., a 60% learning threshold) ensured that all participants adequately learned the experimental material and remained motivated throughout the learning process, it inevitably reduced the variability of declarative memory performance in our study. Yet, as recently highlighted byNebe et al. (2023), low variability, where participants exhibit more similar values, restricts the range of data points and weakens the ability to detect significant associations. It remains thus possible that a less restrictive learning criterion would have led to greater between-subject variability, potentially revealing additional or stronger rsFC associations. That being said, our results still showed valuable interindividual variability in performance scores during the immediate retrieval session (mean ± SD percentage of correct responses: 64 ± 13%; range of scores: min = 28%; max = 83%). Future studies may explore the impact of different learning criteria on the associations between rsFC processes and memory performance. Finally, as this study was based on a cross-sectional sample, it limits our ability to draw definitive conclusions about developmental trajectories and individual differences. Yet, examining declarative learning and memory processes from a developmental perspective may offer an ideal avenue to achieve a deeper understanding of fundamental cognitive processes (Karmiloff‐Smith, 1994). Future studies using experimental designs such as longitudinal investigations across multiple time points would be valuable in assessing the stability and predictive value of theta-band rsFC processes for memory abilities.

Still, our results are in line with previous studies suggesting that theta-band oscillations specifically support the functional basis of long-range brain communication, in particular, between temporal areas and neocortical (mostly prefrontal) areas, required for memory formation in children (Johnson et al., 2022). Moreover, we did not observe a similar relationship between declarative memory performance and rsFC in the alpha and beta frequency bands, strengthening the key specificity of theta-band FC processes for declarative memory.

Unexpectedly, MEG rsFC results did not include (para)hippocampal or medial temporal brain areas, despite their known role in learning and declarative memory processes (Kim, 2011), including those involved in the specific task underused (seeUrbain, De Tiège, et al., 2016). This is also surprising as theta-band oscillations have been repeatedly associated with hippocampal-dependent declarative memory processes (Griffiths et al., 2021;Johnson et al., 2022;Staresina & Wimber, 2019), and hippocampal-related rsFC has been previously highlighted as a possible support for declarative memory performance in young children (Geng et al., 2019;Riggins et al., 2016). Still, these latter studies were ROI hippocampal-based studies, which may have increased the chances of identifying an effect including medial temporal brain regions in past results. Methodologically, it remains possible that the apparent lack of hippocampal involvement in our rsFC results may be due to difficulties in recording magnetic fields emerging from deep sources, at least in the resting state (Wens, 2023). On the other hand, several task-based studies have nowadays demonstrated the ability of MEG to capture hippocampal activity or hippocampal-related functional brain connectivity (López-Madrona et al., 2022;Quraan et al., 2011;Urbain, De Tiège, et al., 2016;Urbain, Vogan, et al., 2016;Urbain et al., 2015,^2017^). The question of whether MEG is sensitive enough to detect spontaneous fluctuations in hippocampal theta oscillations and their connectivity thus remains largely open. Alternatively, we suggest that our results might indicate that rsFC processes do not influence the specific process of binding memory traces, as this function is repeatedly associated with (para)hippocampal brain regions (Geng et al., 2019). Rather, our MEG results indicate that pre-learning rsFC would specifically support sensory input transformation processes, which are underpinned by larger brain networks. Precisely, our data suggest that stronger theta-band rsFC processes would facilitate the subsequent rapid processing and transformation of visuo-semantic information into internal representations during a declarative memory task at school age. Consequently, stronger theta-band rsFC within these networks could lead to a more efficient communication between these brain areas during declarative memory processes and thus to efficient visuo-semantic declarative memory retrieval performance. This idea fits with the hypothesis that resting-state networks would ensure responsiveness to possible future tasks (Deco & Corbetta, 2011) by forming a critical pathway for communication when relevant. However, our study remains correlational and a formal causal relationship between rsFC processes and subsequent memory abilities remains to be investigated in the context of future studies.

Furthermore, the identification of stronger theta-band rsFC as a correlate of better declarative memory performance provides valuable insights into the potential underlying mechanisms contributing to learning difficulties. This could offer potential support for the early and easy (resting-state-dependent) identification of children at risk for declarative learning impairments, particularly in the context of low cognitive load protocols (Riggins et al., 2016;Uddin, 2010), and possible insights regarding clinical routines and procedures.

## Data Availability

The data that support the findings of this study are available on request from the corresponding author and after acceptance by institutional authorities (Hôpital Universitaire de Bruxelles and Université libre de Bruxelles). The data are not publicly available due to ethical restrictions.
